# Influencing Factors of Recurrence of Nonvalvular Atrial Fibrillation after Radiofrequency Catheter Ablation and Construction of Clinical Nomogram Prediction Model

**DOI:** 10.1155/2022/8521735

**Published:** 2022-03-15

**Authors:** Zhong-bao Ruan, Hong-xia Liang, Fei Wang, Ge-cai Chen, Jun-guo Zhu, Yin Ren, Li Zhu

**Affiliations:** ^1^Department of Cardiology, Jiangsu Taizhou People's Hospital, Taizhou 225300, China; ^2^Dalian Medical University, Dalian 116044, China

## Abstract

**Purpose:**

This study sought to investigate the predictive factors for atrial fibrillation (AF) recurrence in patients after radiofrequency ablation (RFCA) and construct a nomogram prediction model for providing precious information of ablative strategies.

**Methods:**

A total of 221 patients with AF who underwent RFCA were enrolled. Univariate and multivariate Cox regression were used to screen the predictors of recurrence. The receiver operating characteristic (ROC) curve and the Kaplan–Meier (K–M) curve were drawn to analyze the value of predictors. The nomogram model was further constructed to predict the recurrence of AF in patients after RFCA.

**Results:**

There were 59 cases of AF recurrence after RFCA. Monocyte count/high-density lipoprotein cholesterol (MHR), AF course (COURSE), coronary heart disease (CHD), and AF type (TYPE) were the independent risk factors for predicting AF recurrence after RFCA. Accordingly, a nomogram prediction model based on MHR, COURSE, CHD, and TYPE was constructed with a C-index of 0.818 (95% CI: 0.681∼0.954), while the C-index of verification was 0.802 (95% CI: 0.658∼0.946).

**Conclusions:**

Preoperative MHR, COURSE, CHD, and TYPE were independent risk factors for predicting recurrence of AF after RFCA. The nomogram model based on MHR, COURSE, CHD, and TYPE can be used to predict the recurrence of AF after RFCA accurately and individually.

## 1. Introduction

Atrial fibrillation (AF) is the most common persistent arrhythmia in clinics. Radiofrequency catheter ablation (RFCA) has gradually become the most effective method to restore sinus rhythm and improve quality of life in patients with AF [1]. However, although the techniques and technologies of RFCA have improving and have been associated with a higher clinical success rate, previous clinical studies have shown that the recurrence of AF after single RFCA varied from 11% to 29% and 7% to 24% after repeated RFCA in paroxysmal AF during 5 years follow-up, while up to 70% in persistent AF [2–4]. Therefore, it is very important to identify the predictors of AF recurrence after RFCA, which will help to improve the successful rate of RFCA and guide clinical practice.

At present, many studies have been performed and limited to looking for the factors affecting atrial remodeling and involving the pathogenesis of AF as an index to predict the recurrence of AF [5, 6]. Meanwhile, the predictive value of these factors for the recurrence of AF after RFCA was still controversial. At the same time, there were few relevant studies focusing on the construction of the prediction model for AF recurrence after RFCA. In this study, univariate and multivariate Cox regression analysis were used to screen the risk factors for predicting the recurrence of AF after RFCA, and a nomogram prediction model based on screening was constructed to individually evaluate the risk of recurrence of AF.

## 2. Methods

### 2.1. Study Population

This study included 221 patients with symptomatic AF who underwent RFCA at Jiangsu Taizhou People's Hospital from January 2017 to January 2019, of whom 139 had paroxysmal AF, 82 had persistent AF. The mean age was 61.93 ± 9.72 years, and 131 (59.3%) of the participants were male. All patients were included with the criteria as follows: AF was confirmed by history, ECG or Holter, and the treatment with at least one antiarrhythmic drug was ineffective and willing to undergo RFCA. The type of AF was classified according to the ESC Guidelines on AF 2020 [7]. Exclusion criteria included the following: history of clinical and echocardiographic evidence of chronic heart failure, New York Heart Association (NYHA) class III–IV, moderate and severe valvular heart disease, thyroid dysfunction, contraindications for anticoagulation, left atrium and/or left atrial appendage with thrombus, and expected survival of less than 1 year. The study was approved by the Ethics Committee of Jiangsu Taizhou People's Hospital. Written informed consent of all patients was obtained from all patients before the procedure.

### 2.2. RFCA Procedure and Postprocedural Management

The RFCA procedure was performed under mild sedation with midazolam. Following transseptal puncture, intravenous heparin was used by femoral vein access to achieve an activated clotting time (ACT) > 300 s. Then, pulmonary vein isolation (PVI) or PVI + linear ablation and/or left atrial complex fragmentation potential ablation was performed under the guidance of a three-dimensional mapping system (CARTO 3, Biosense Webster, Inc.). In brief, a PVI ablation was undergone in all patients. The endpoints of PVI were defined as the blocks of bidirectional pacing. If AF was sustained after PVI, electrical cardioversion was performed. For patients with persistent AF, if AF was sustained after PVI, electrical cardioversion was also performed. Then, an ablation of the roofline, posteriorinferior line, anterior line, or complex fractionated electrograms was conducted. Anticoagulant and antiarrhythmic drugs were taken for 3 months after procedure.

### 2.3. Clinical Data

In this study, the clinical characteristics, medical history, echocardiographic features, medication, and CHA_2_DS_2_-VASc score of each patient were recorded. About 10 ml of peripheral blood was drawn for blood routine, biochemical, and coagulation function tests from each participant 24 hours after admission. A monocyte count-to-HDL cholesterol ratio (MHR) was measured. All patients were followed up at 3, 6, and 12 months and later every 6 months after ablation. 12-lead ECG and 24-hour Holter monitoring were routinely performed simultaneously. Patients experiencing palpitations and other discomfort were required to undergo repeat 24- to 72-hour Holter monitoring. Recurrence of AF was defined as AF or atrial flutter or atrial tachycardia lasting for more than 30 s documented by 12-lead ECG or 24-hour Holter monitoring after a 3-month blank period of RFCA.

### 2.4. Statistical Analysis

SPSS 24.0 statistical software was used for data analysis. Continuous variables conforming to the normal distribution were described in the form of mean ± standard deviation, while continuous variables not conforming to the normal distribution were described in the form of median (quad interval). Categorical variables are reported as counts and percentages. The risk factors of recurrence were analyzed by univariate analysis and multivariate Cox risk model. The cumulative survival rate was calculated by the Kaplan–Meier (K–M) curve. The receiver operating characteristic (ROC) curve was used to evaluate the value of risk factors in predicting recurrence. The nomogram method was used to construct the risk prediction model of recurrence after AF ablation. *p* < 0.05 was statistically significant.

## 3. Results

### 3.1. Patient Characteristics and Single Factor Analysis Results of AF Recurrence after RFCA

There were 59 cases of AF recurrence after RFCA. Baseline group characteristics are presented in Table 1. In single factor analysis, there was no significant difference in age, gender, hypertension, diabetes, stroke, white blood cell count, neutrophil count, lymphocyte count, BMI, CHA_2_DS_2_-VASC score, LDL-C, LAD, and lipoprotein level between the recurrence group and the nonrecurrence group (*p* > 0.05). However, there were significant differences in coronary heart disease (CHD), duration of AF (COURSE), type of atrial fibrillation (TYPE), uric acid, LVEF, and MHR (*p* < 0.05), as shown in detail in Table 1.

### 3.2. Multiple Cox Regression Analysis of AF Recurrence after RFCA

As shown in Table 2, MHR, COURSE, CHD, and TYPE were the independent risk factors for recurrence of AF after RFCA. The regression coefficient (*β*), relative risk (RR), and 95% confidence interval (CI) of MHR were 2.529, 12.546, and (4.641, 33.915), respectively. While 1.118, 3.060, and (1.643, 5.700) were of CHD, 0.006, 1.006, and (1.002, 1.009) were of COURSE, and 0.817, 2.263, and (1.205, 4.250) were of TYPE.

### 3.3. Analysis of the Independent Risk Factors for AF Recurrence with the ROC Curve and K–M Curve

**Figure 1:**
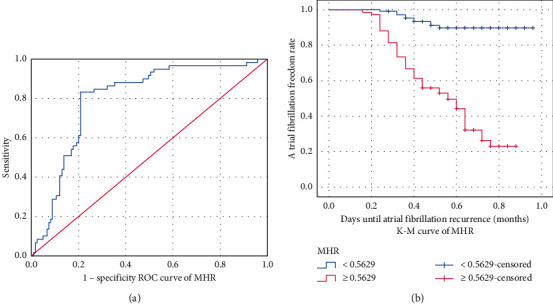
Analysis of MHR for predicting AF recurrence after RFCA with ROC curve (a) and K–M curve (b).

The AUC of MHR for predicting AF recurrence was 0.800 and the cutoff value was 0.5629, with a sensitivity of 83.1% and specificity of 79.2% (Figure 1(a)). According to the cutoff value of MHR, the average recurrence time of AF was 22.579 months (95% CI: (21.738, 23.419)) when MHR <0.5629 and 13.852 months (95% CI: (12.466, 15.238)) when MHR ≥0.5629 (Table 3). According to the cutoff value of MHR, the K–M survival curve was drawn and showed that the risk of recurrence of AF was higher when MHR ≥0.5629 (Figure 1(b)), which suggested that MHR was an independent risk factor for late recurrence of AF after RFCA.

**Figure 2:**
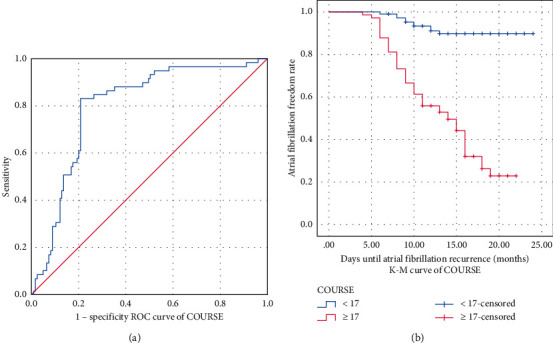
Analysis of COURSE for predicting AF recurrence after RFCA with ROC curve (a) and K–M curve (b).

The AUC of COURSE for predicting AF recurrence was 0.790 and the cutoff value was 17 months, with a sensitivity of 76.3% and specificity of 75.2% (Figure 2(a)). According to the cutoff value of COURSE, the average recurrence time of AF was 21.917 months (95% CI: 20.905∼22.928) when COURSE <17 months and 14.193 months (95% CI: 12.668∼15.717) when COURSE ≥17 months (Table 4). According to the cutoff value of COURSE, a K–M survival curve was drawn and showed that the risk of late recurrence of AF was higher when COURSE ≥17 months (Figure 2(b)), which suggested that COURSE was an independent risk factor for late recurrence of AF after RFCA.

**Figure 3:**
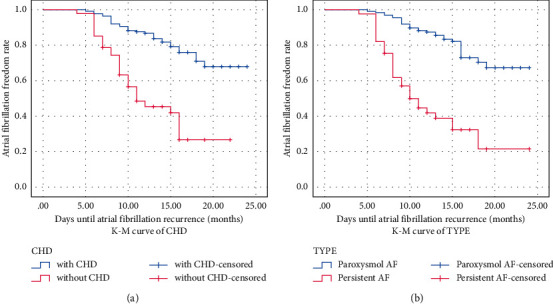
Analysis of CHD (a) and AF type (b) for predicting AF recurrence after RFCA with K–M curve.

Survival analysis of AF recurrence showed that the average recurrence time was 20.464 months (95% CI: 19.359∼21.570) in patients without CHD, while 13.414 months (95% CI: 11.546∼15.281) in patients combined with CHD (Table 5). According to whether AF patient was combined with CHD, a K–M survival curve was drawn and indicated that AF patients combined with CHD have higher risk of late recurrence of AF after RFCA (Figure 3(a)). Of those 82 persistent AF patients, recurrence occurred in 29 (29 out of 82; 35.4%). On the contrary, recurrence was observed in 30 patients with paroxysmal AF (30 out of 139; 21.6%). There was a significant difference of the recurrence percentage between persistent AF and paroxysmal AF. A survival analysis of AF recurrence according to TYPE was performed and showed that the average recurrence time of paroxysmal AF was 20.545 months (95% CI: 18.829∼21.019), while 13.149 months (95% CI: 10.867∼14.470) in persistent AF (Table 6). A K–M survival curve was drawn according to TYPE and indicated that patients with persistent AF had a higher risk of late recurrence of AF after RFCA (Figure 3(b)).

### 3.4. Construction and Verification of a Nomogram Model for Predicting the Risk of AF Recurrence after RFCA

**Figure 4:**
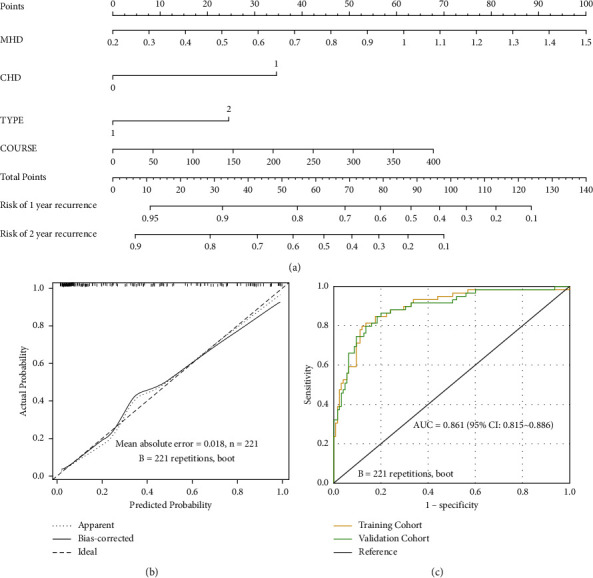
Construction and verification of a nomogram model for predicting the risk of AF recurrence after RFCA. (a) Nomogram model of recurrence risk after RFCA for AF patients. (b) Calibration curve of nomogram model Bootstrap after self-sampling. (c) ROC curve of nomogram model bootstrap after self-sampling.

Accordingly, a nomogram model based on MHR, COURSE, CHD, and TYPE was constructed to predict the recurrence risk of AF in patients after RFCA. As shown in the nomogram model (Figure 4(a)), with the extension of COURSE, increase of MHR, and combination with CHD and persistent atrial fibrillation, the nomogram score gradually increased and the risk degree increased accordingly, which indicated that the survival time gradually decreased. In the nomogram model, the scores of MHR, COURSE, combined with CHD and TYPE were 100, 70, 35, and 28, respectively. The total score obtained by calculating all risk factors could be used to estimate the risk of recurrence of atrial fibrillation 1 year and 2 years after RFCA in patients with multiple risk factors, with a C-index of 0.818 (95% CI: 0.681∼0.954), while C-index of verification was 0.802 (95% CI: 0.658∼0.946) (Figure 4(b)). The AUC was 0.861 (95% CI: 0.815∼0.886), the sensitivity was 79.21%, and the specificity was 82.36%, which indicated that the model had good accuracy and differentiation (Figure 4(c)).

## 4. Discussion

Studies have shown that monocyte content has been associated with the development of cardiovascular diseases such as atherosclerosis and AF. In the atrium, high levels of TNF, TGF, interleukin-2 (IL-2), and IL-6 were secreted by monocytes [5, 6]. IL-6 is associated with the recurrence of AF after ablation [7]. Abnormity of action potential mediated by IL-2 is associated with shortened cardiac electrical activity time. TNF can interfere with calcium homeostasis, shorten the duration of action potential, and increase myocardial apoptosis and myolysis. TNF and TGF are also involved in the activation of fibroblasts, and the activated fibroblasts convert to myofibroblasts, leading to increased atrial fibrosis [8, 9]. High-density lipoprotein cholesterol (HDL-C), synthesized by liver cells, has the functions of being anti-inflammatory and reducing oxidative stress. Low levels of HDL-C may attenuate anti-inflammatory and antioxidative effects, promote the development of atrial fibrosis, and increase the susceptibility to AF [10]. MHR, a ratio of monocyte count to HDL, is a novel serological marker of inflammatory and oxidative stress. MHR was found to be an independent risk factor for recurrent paroxysmal atrial fibrillation after cryoablation, with a sensitivity of 89% and specificity of 54% [11]. The results of this study showed that MHR was an independent risk factor for late recurrence of nonvalvular AF after catheter ablation, with a cutoff value of 0.5629, corresponding to a sensitivity of 83.1% and a specificity of 79.2%.

The association between CHD and AF has been reported. Previous experimental animal studies showed that acute myocardial ischemia is associated with higher atrial vulnerability contributing to AF development by inducing the decreased inward of L-type Ca^2+^, increased outflow of K^+^, and reduction of refractory period [12]. CHD can also directly promote the development of AF by affecting reentry formation, focal ectopic activity, and neural remodeling [13]. In the present study, 30 patients (50.84%) with CHD were observed in the recurrence group, while only 23 patients (14.2%) in 162 nonrecurrence group had CHD. There was a significant difference between the recurrence group and the nonrecurrence group. Meanwhile, the average recurrence time of AF in patients with CHD after catheter ablation was significantly shorter than that in patients without CHD, which suggested that CHD was an independent risk factor for recurrence after AF ablation. Our study results consisted of previous reports.

Studies have shown that there are higher rates of AF freedom after RFCA in paroxysmal AF than in persistent AF [14, 15]. In the present study, of those 82 persistent AF patients, recurrence occurred in 29 (29 out of 82; 35.4%), while in 30 patients with paroxysmal AF (30 out of 139; 21.6%). There was a significant difference of the recurrence percentage between persistent AF and paroxysmal AF. The average time of AF recurrence in patients with paroxysmal AF was 20.545 months, whereas it was 13.149 months in persistent AF. Our results indicated that AF type was an independent risk factor for AF recurrence after catheter ablation.

The AF COURSE contributed greatly to recurrence after ablation; the longer the course time, the higher the recurrence rate [16]. In this study, according to the cutoff value of 17 months, the sensitivity and specificity of COURSE for predicting AF recurrence were 76.3% and 75.2%, respectively. When COURSE of AF was less than 17 months, the average time of AF recurrence was 21.917 months, while 14.193 months when the COURSE of AF was more than 17 months, which indicated that the COURSE of AF was an independent risk factor for late recurrence of AF.

The nomogram is a kind of graphical model for estimating specific outcomes or survival in association with certain risk factors. Although RFCA for AF has achieved a high rate of AF freedom, recurrence is still unavoidable. Meanwhile, it is also very difficult to assess the risk factors of recurrence for every patient with AF after RFCA. It is also very difficult to identify those patients who are prone to recurrence in the absence of a prediction model. In this study, a nomogram model of AF recurrence risk was constructed based on the four independent risk factors including MHR, CHD, COURSE, and TYPE. The verification of the nomogram model of AF recurrence risk showed that the AUC was 0.861 (95% CI: 0.815∼0.886), sensitivity was 79.21%, and specificity was 82.36%, with a the C-index of 0.818 (95% CI: 0.681∼0.954), while C-index of verification was 0.802 (95% CI: 0.658∼0.946), which suggested that the nomogram model of AF recurrence has good accuracy and differentiation for screening the recurrence risk in AF patients after RFCA. Meanwhile, our data suggested that patients with higher MHR, longer COURSE, CHD, and persistent AF had a higher risk of recurrence. We can perform a personalized risk assessment and guide the optimal treatment for patients with a high risk of AF recurrence according to the nomogram model.

## 5. Limitations of the Study

The present study was a single-center study and had a small sample size, so the representativeness of the research sample was relatively insufficient. A multicenter study with larger samples should be conducted to further enhance the results. Furthermore, this nomogram model needs further external validation.

## Figures and Tables

**Table 1:** Patient characteristics and comparison of postoperative recurrence.

Variable	All (*n* = 221)	AF recurrence group (*n* = 59)	Nonrecurrence group (*n* = 162)	*p* value	OR
Age, years	61.93 ± 9.72	61.64 ± 9.88	62.04 ± 9.88	0.530	0.991
BMI (kg/m^2^)	25.11 ± 3.30	25.06 ± 3.31	25.14 ± 3.35	0.990	0.999
Male, *n* (%)	131 (59.3)	40 (67.8)	91 (56.2)	0.095	1.592
Hypertension, *n* (%)	119 (53.8)	32 (54.2)	87 (53.7)	0.757	0.922
DM, *n* (%)	25 (11.3)	6 (10.1)	19 (11.7)	0.872	0.940
Smoking, *n* (%)	53 (23.9)	15 (25.4)	38 (23.5)	0.718	0.963
Stroke/TIA history, *n* (%)	29 (13.1)	9 (15.3)	20 (12.3)	0.243	0.666
CHD, *n* (%)	53 (23.9)	30 (50.8)	23 (14.2)	0.003	2.332
COURSE (months)	28.36 ± 28.34	48.68 ± 34.54	16.71 ± 15.25	≤0.001	3.007
TYPE (persistent AF), *n* (%)	50 (22.6)	29 (49.2)	21 (13)	≤0.001	3.379
CHA2DS2-VASC score	2.39 ± 1.49	2.33 ± 1.45	2.43 ± 1.54	0.370	0.677
UA (mmol/l)	353.27 ± 94.96	377.61 ± 89.26	338.67 ± 96.72	0.022	1.003
Creatinine (*μ*mol/L)	73.25 ± 18.69	71.84 ± 18.35	73.24 ± 19.85	0.324	0.662
HDL (mmol/L)	1.45 ± 0.57	1.41 ± 0.58	1.47 ± 0.58	0.607	0.891
LDL (mmol/l)	2.25 ± 0.52	2.29 ± 0.55	2.22 ± 0.51	0.784	0.954
LAD (mm)	43.42 ± 5.92	44.77 ± 6.08	42.59 ± 5.76	0.181	1.029
MHR	0.61 ± 0.23	0.73 ± 0.21	0.52 ± 0.21	≤0.001	9.518
Follow-up, months	12.02 ± 3.66	11.61 ± 3.45	12.26 ± 384	0.237	0.621

**Table 2:** Multiple Cox regression analysis of AF recurrence after RFCA.

	Variable	*β*	*p*	RR	95% CI	
					Lower	Upper
Steps	MHR	2.529	≤0.001	12.546	4.641	33.915
	CHD	1.118	≤0.001	3.060	1.643	5.700
	CUORSE	0.006	0.001	1.006	1.002	1.009
	TYPE	0.817	0.011	2.263	1.205	4.250

**Table 3:** The average recurrence time of AF after RFCA according to MHR stratification.

MHR	The average recurrence time (months)	Standard error	95% CI	
			Lower	Upper
<0.5629	22.579	0.429	21.738	23.419
≥0.5629	13.852	0.707	12.466	15.238

**Table 4:** The average recurrence time of AF after RFCA according to COURSE stratification.

COURSE	The average recurrence time (months)	Standard error	95% CI	
			Lower	Upper
<17	21.917	0.516	20.905	22.928
≥17	14.193	0.778	12.668	15.717

**Table 5:** The average recurrence time of AF after RFCA in patients with or without CHD.

CHD	The average recurrence time (months)	Standard error	95% CI	
			Lower	Upper
Without	20.464	0.564	19.359	21.570
With	13.414	0.953	11.546	15.281

**Table 6:** The average recurrence time of AF after RFCA according to TYPE.

Type	The average recurrence time (months)	Standard error	95% CI	
			Lower	Upper
Paroxysmal AF	20.545	0.548	19.472	21.619
Persistent AF	13.149	1.153	10.889	15.409

## Data Availability

The data used to support the findings of this study are available from the corresponding author upon request.
